# Supplementary material for: Tumour-informed liquid biopsies to monitor advanced melanoma patients under immune checkpoint inhibition
Source: Nat Commun. 2024 Oct 9;15:8750. doi: 10.1038/s41467-024-52923-0 (PMC11464631; doi:10.1038/s41467-024-52923-0)
Supplement: Supplementary file 1 — Supplementary Information [file 41467_2024_52923_MOESM1_ESM.pdf]

Supplement

Supplementary Table 1: Error rates for different error correction levels. 1-fold - no duplicate, 2-fold - one duplicate, 3-fold - two duplicates, 4-fold - three duplicates. Source data are provided as a Source Data file.

Error rate	UMI copies available for error correction ("correction level")			
	1-fold	2-fold	3-fold	4-fold or higher
mean	$1.76x10^{-3}$	$2.47x10^{-4}$	$9.00x10^{-5}$	$3.17x10^{-5}$
min	$1.89x10^{-4}$	$4.58x10^{-5}$	$1.45x10^{-5}$	$1.03x10^{-5}$
max	$3.97x10^{-3}$	$1.24x10^{-3}$	$6.73x10^{-4}$	$9.68x10^{-5}$

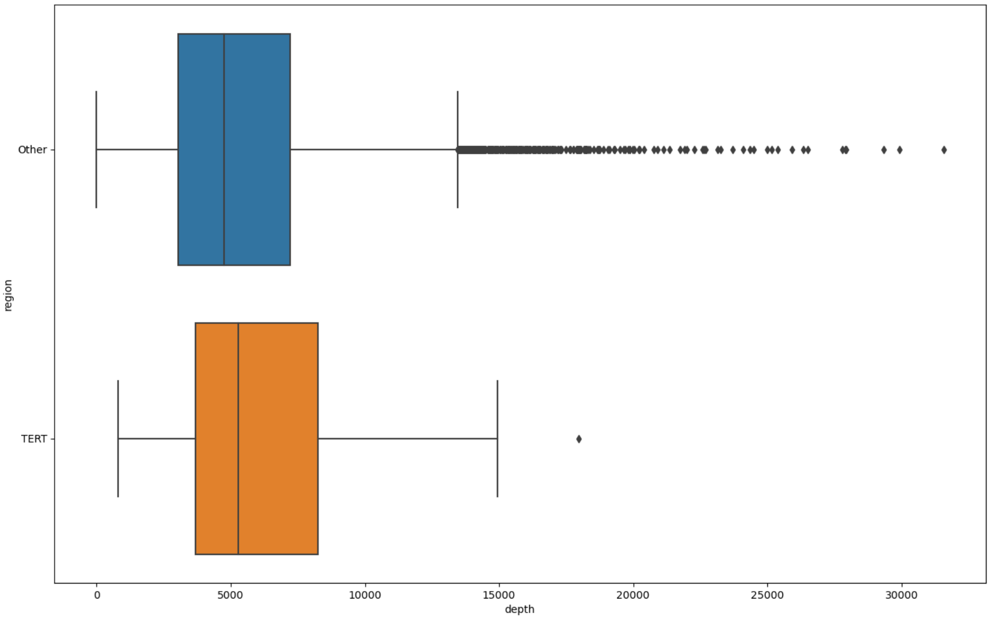

Supplementary Fig. 1: Sequencing depth for *TERT* promoter variants. Distribution of the sequencing depth for *TERT* promoter variants in samples of 26 patients compared to all variants of all patients. There is no difference in the distribution of sequencing depth for promoter variants in *TERT* compared to other variants. Source data are provided as a Source Data file.

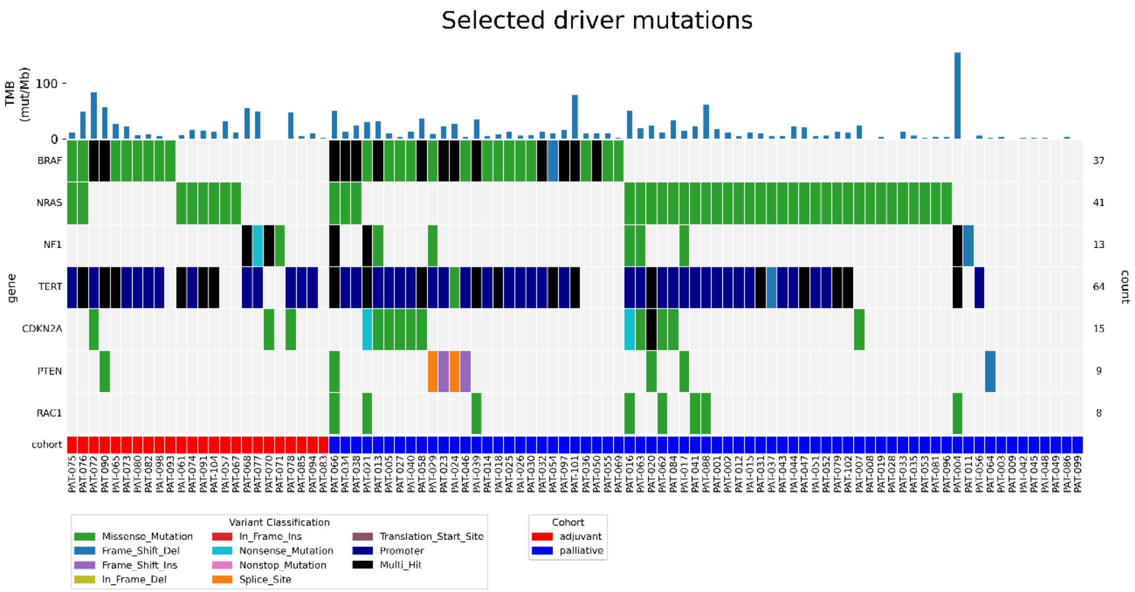

Supplementary Fig. 2: Oncoplot with selected driver mutations. Oncoplot with mutations in selected driver genes in our patient cohort. The oncoplot includes hotspot and non-hotspot mutations. Six patients were excluded from subsequent analysis, since their liquid biopsies failed quality control. Source data are provided as a Source Data file.

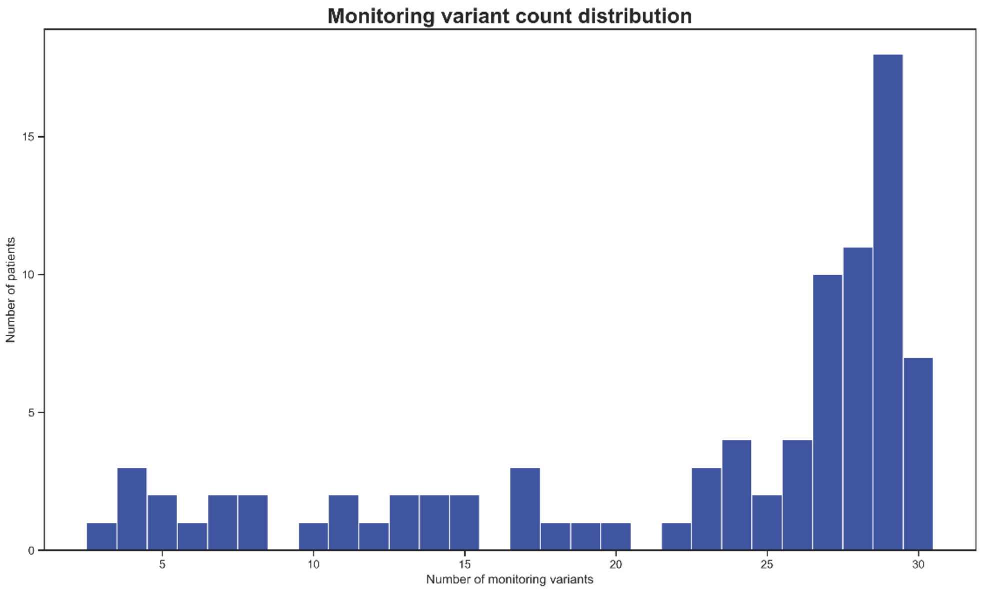

Supplementary Fig. 3: Monitoring variant count distribution. Histogram of the number of somatic mutations per patient which were designed for therapy monitoring or relapse detection. Few patients had less variants which was related to lower DNA-quality, lower sequencing quality or lower number of detected tumour mutations. Source data are provided as a Source Data file.

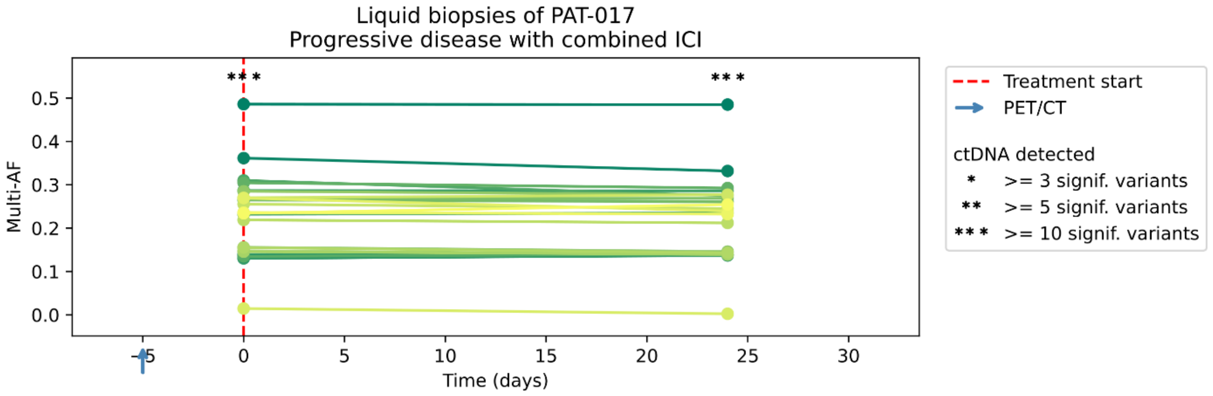

Supplementary Fig. 4: Patient with progressive disease and high AFs. Example of a patient with progressive disease and high AFs of tumour variants in his plasma. The AFs remain stable under treatment despite the clinical observation of progressive disease, which may be explained by saturation of cell-free DNA with ctDNA. Source data are provided as a Source Data file.

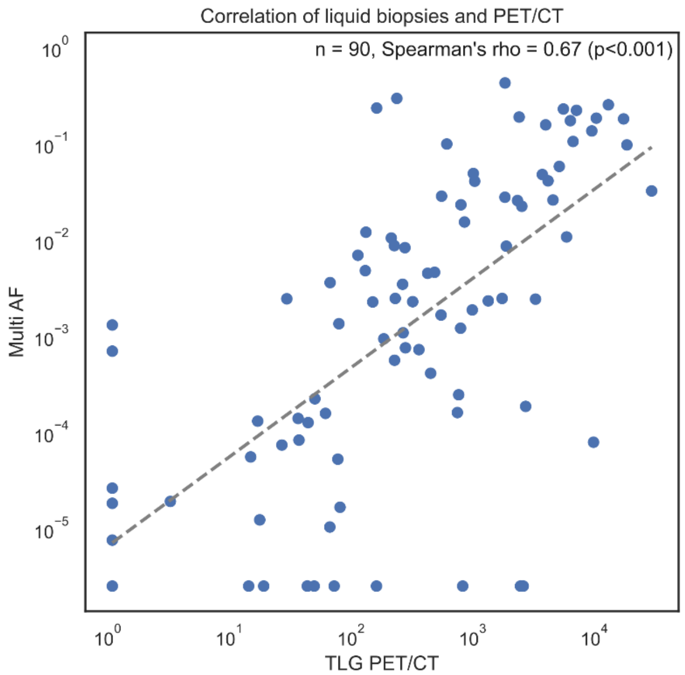

Supplementary Fig. 5: Correlation AF and TLG. Correlation of mutation allele fractions in plasma with TLG (ml) for the palliative cohort. TLG – ml. Source data are provided as a Source Data file.

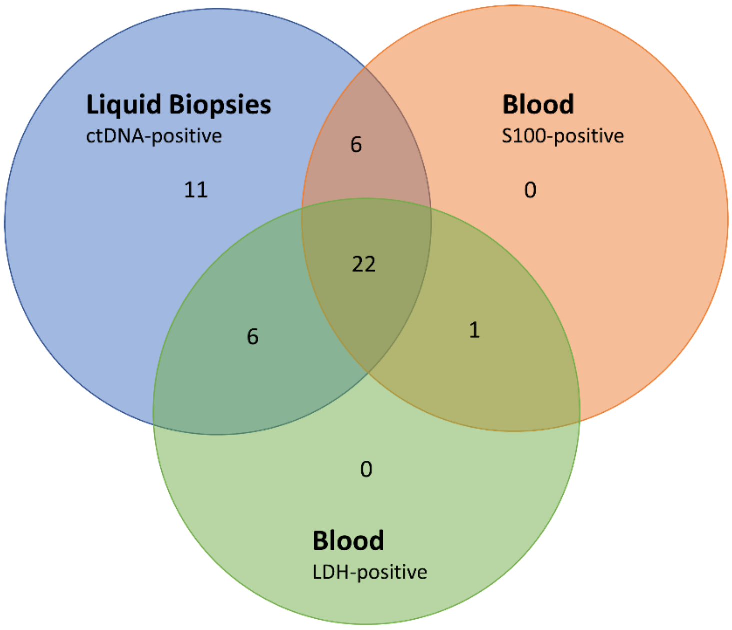

Supplementary Fig. 6: Concordance of different biomarkers at T0. The Venn-diagram shows the overlap of biomarker-positive samples +/- 7 days of T0 in the group of advanced melanoma patients under combined ICI. Only patients with results for all biomarkers were included (n=50). While all three biomarkers were concordant for the majority of patients, it can be seen that liquid biopsies detected additional patients compared to S100 or LDH with only one patient missed and only detected by S100 and LDH. ctDNA samples: ≥ 3 variants with sufficient sequencing depth, ctDNA-positive: ≥ 3 detected variants. LDH-positive > 250 ug/l. S100-positive ≥ 0.1 ug/l. Source data are provided as a Source Data file.

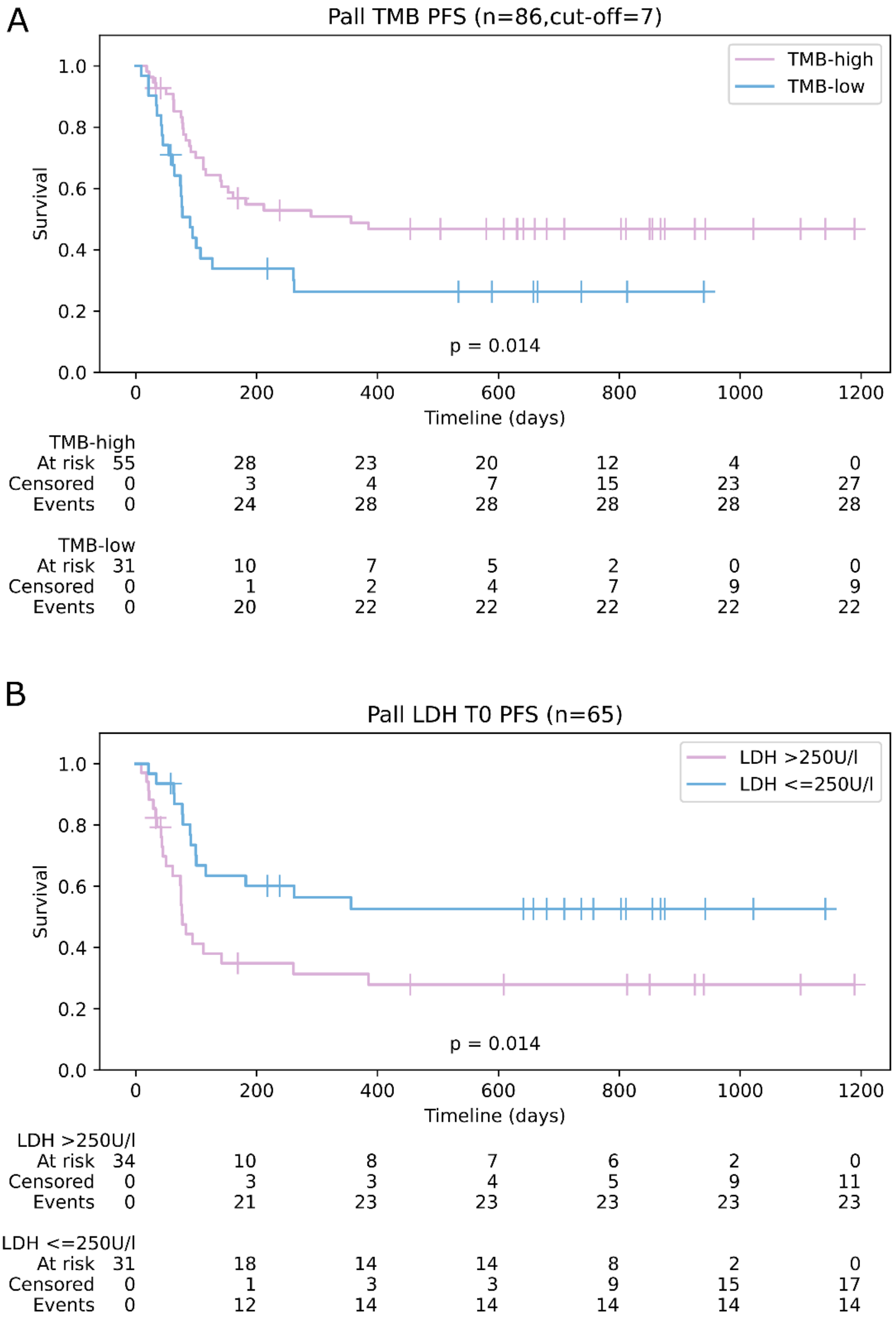

Supplementary Fig. 7: Additional survival curves for PFS and TMB/LDH. Subpanel A shows PFS and TMB from tumour sequencing for the palliative combined ICI and adjuvant cohort, subpanel B PFS for LDH at time point 0 for the palliative combined ICI cohort. TMB-low ≤ 7 variants/Mbp, TMB-high > 7 variants/Mbp. Source data are provided as a Source Data file.

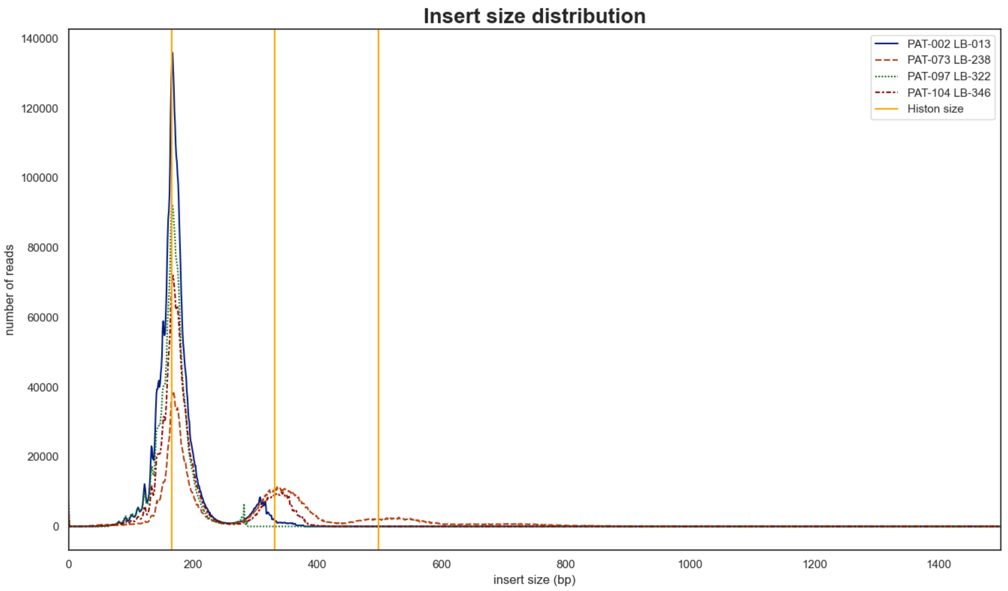

Supplementary Fig. 8: Insert size distribution of fragments in selected LBs. Distribution plot of the insert size of 4 randomly selected samples (mean insert size between 168bp and 289bp). The peaks correspond to the expected lengths of multiples of the size of DNA coiled around a histone. The vast majority of fragments stems from fragments of 160-170bp (one histone), small fractions correspond to DNA molecules coiled around two (appr. 320bp) or three histones (appr. 480bp). The number of fragments beyond the 480bp peaks is very low, indicating little genomic DNA from blood cells. The existence of very long genomic fragments cannot be ruled out. However, mosaic mutations in white blood cells are mostly found in a small set of CHIP (Clonal haematopoiesis of indeterminate potential) genes such as *DNMT3* and *TET2* genes, and are highly unlikely to effect the 30 sites monitored in our patients. Therefore, the tumour-informed monitoring method can, other than liquid biopsy using larger gene panels, tolerate small contamination by genomic DNA from white blood cells.
