# Supplementary material for: Tumour-informed liquid biopsies to monitor advanced melanoma patients under immune checkpoint inhibition
Source: Nat Commun. 2024 Oct 9;15:8750. doi: 10.1038/s41467-024-52923-0 (PMC11464631; doi:10.1038/s41467-024-52923-0)
Supplement: Supplementary file 3 — Reporting Summary [file 41467_2024_52923_MOESM3_ESM.pdf]

nature portfolio

Corresponding author(s): Andrea Forschner, Christopher Schroeder

Last updated by author(s): Jun 12, 2024

# Reporting Summary

Nature Portfolio wishes to improve the reproducibility of the work that we publish. This form provides structure for consistency and transparency in reporting. For further information on Nature Portfolio policies, see our Editorial Policies and the Editorial Policy Checklist.

## Statistics

For all statistical analyses, confirm that the following items are present in the figure legend, table legend, main text, or Methods section.

| n/a | Confirmed | |
|---|---|---|
| ☐ | ☒ | The exact sample size (*n*) for each experimental group/condition, given as a discrete number and unit of measurement |
| ☐ | ☒ | A statement on whether measurements were taken from distinct samples or whether the same sample was measured repeatedly |
| ☐ | ☒ | The statistical test(s) used AND whether they are one- or two-sided<br>*Only common tests should be described solely by name; describe more complex techniques in the Methods section.* |
| ☐ | ☒ | A description of all covariates tested |
| ☐ | ☒ | A description of any assumptions or corrections, such as tests of normality and adjustment for multiple comparisons |
| ☐ | ☒ | A full description of the statistical parameters including central tendency (e.g. means) or other basic estimates (e.g. regression coefficient) AND variation (e.g. standard deviation) or associated estimates of uncertainty (e.g. confidence intervals) |
| ☐ | ☒ | For null hypothesis testing, the test statistic (e.g. *F*, *t*, *r*) with confidence intervals, effect sizes, degrees of freedom and *P* value noted<br>*Give P values as exact values whenever suitable.* |
| ☒ | ☐ | For Bayesian analysis, information on the choice of priors and Markov chain Monte Carlo settings |
| ☒ | ☐ | For hierarchical and complex designs, identification of the appropriate level for tests and full reporting of outcomes |
| ☐ | ☒ | Estimates of effect sizes (e.g. Cohen's *d*, Pearson's *r*), indicating how they were calculated |

*Our web collection on statistics for biologists contains articles on many of the points above.*

## Software and code

Policy information about availability of computer code

| | |
|---|---|
| Data collection | Data was collected using Python scripts, MS Excel and exports of the patient's electronic health records. |
| Data analysis | We developed a new analysis tool, umiVar (unpublished, https://github.com/imgag/umivar) to enable efficient sequencing error correction and accurate detection of ultra-low fractions of ctDNA using UMI-barcoded reads. For raw read processing and mapping the MegSAP pipeline was applied (https://github.com/imgag/megSAP). |

For manuscripts utilizing custom algorithms or software that are central to the research but not yet described in published literature, software must be made available to editors and reviewers. We strongly encourage code deposition in a community repository (e.g. GitHub). See the Nature Portfolio guidelines for submitting code & software for further information.

## Data

Policy information about availability of data

All manuscripts must include a data availability statement. This statement should provide the following information, where applicable:

- Accession codes, unique identifiers, or web links for publicly available datasets
- A description of any restrictions on data availability
- For clinical datasets or third party data, please ensure that the statement adheres to our policy

*Provide your data availability statement here.*

## Research involving human participants, their data, or biological material

Policy information about studies with human participants or human data. See also policy information about sex, gender (identity/presentation), and sexual orientation and race, ethnicity and racism.

| | |
|---|---|
| Reporting on sex and gender | No sex- and gender-based analyses have been performed. |
| Reporting on race, ethnicity, or other socially relevant groupings | We did not collect or use information on race, ethnicity or any other socially relevant groupings in this study. |
| Population characteristics | See above. |
| Recruitment | We included all melanoma patients who started combined ICI with ipilimumab and nivolumab between July 2019 and May 2021, of whom tumour tissue was available (Group 1). From September 2020 onwards, adjuvant ICI patients were also included via an amendment in order to evaluate ctDNA as biomarker for the detection of relapse (Group 2). |
| Ethics oversight | The study was approved by the local ethical review board (project ID: 196/2019BO2). |

Note that full information on the approval of the study protocol must also be provided in the manuscript.

# Field-specific reporting

Please select the one below that is the best fit for your research. If you are not sure, read the appropriate sections before making your selection.

☒ Life sciences ☐ Behavioural & social sciences ☐ Ecological, evolutionary & environmental sciences

For a reference copy of the document with all sections, see nature.com/documents/nr-reporting-summary-flat.pdf

# Life sciences study design

All studies must disclose on these points even when the disclosure is negative.

| | |
|---|---|
| Sample size | This exploratory study included 104 patients. |
| Data exclusions | Four patients did not start with ICI because of disease progression or retracted consent, tumour sequencing was not possible in 7 patients, and plasma samples were insufficient for an additional 6 patients. |
| Replication | n/a |
| Randomization | n/a |
| Blinding | The study was not blinded. |

# Reporting for specific materials, systems and methods

We require information from authors about some types of materials, experimental systems and methods used in many studies. Here, indicate whether each material, system or method listed is relevant to your study. If you are not sure if a list item applies to your research, read the appropriate section before selecting a response.

## Materials & experimental systems

| n/a | Involved in the study | |
|---|---|---|
| ☒ | ☐ | Antibodies |
| ☒ | ☐ | Eukaryotic cell lines |
| ☒ | ☐ | Palaeontology and archaeology |
| ☒ | ☐ | Animals and other organisms |
| ☐ | ☒ | Clinical data |
| ☒ | ☐ | Dual use research of concern |
| ☒ | ☐ | Plants |

## Methods

| n/a | Involved in the study | |
|---|---|---|
| ☒ | ☐ | ChIP-seq |
| ☒ | ☐ | Flow cytometry |
| ☒ | ☐ | MRI-based neuroimaging |

## Clinical data

Policy information about clinical studies

All manuscripts should comply with the ICMJE guidelines for publication of clinical research and a completed CONSORT checklist must be included with all submissions.

| | |
|---|---|
| Clinical trial registration | DRKS00017724 |
| Study protocol | Observational study, please refer to the methods section of the manuscript |
| Data collection | S. recruitment above |
| Outcomes | Overall survival, progression free survival. Differences between responders and non-responders to therapy based on ctDNA levels. |

## Plants

| | |
|---|---|
| Seed stocks | n/a |
| Novel plant genotypes | n/a |
| Authentication | n/a |
