# Supplementary material for: Tumour-informed liquid biopsies to monitor advanced melanoma patients under immune checkpoint inhibition
Source: Nat Commun. 2024 Oct 9;15:8750. doi: 10.1038/s41467-024-52923-0 (PMC11464631; doi:10.1038/s41467-024-52923-0)
Supplement: Supplementary file 4 — Description of Additional Supplementary Files [file 41467_2024_52923_MOESM4_ESM.pdf]

Description of Additional Supplementary Files

File Name: Supplementary Data 1

Description: This file contains a list of variants that were used to study ctDNA of patients in the PET/LIT-study.
